# Repair of segmental bone defect using tissue engineered heterogeneous deproteinized bone doped with lithium

**DOI:** 10.1038/s41598-021-84526-w

**Published:** 2021-03-01

**Authors:** Jun Li, Wenzhao Wang, Mingxin Li, Lei Liu

**Affiliations:** Department of Orthopedics, National Clinical Research Center for Geriatrics, West China Hospital, Sichuan University, 37# Wainan Guoxue Road, Chengdu, 610041 Sichuan China

**Keywords:** Mesenchymal stem cells, Musculoskeletal system

## Abstract

Lithium have been shown to play an important role in improving the osteogenic properties of biomaterials. This study aims to explore the osteogenic improvement effect of tissue engineered heterogeneous deproteinized bone (HDPB) doped with lithium, and evaluate their effectiveness in the healing of bone defects. Bone marrow mesenchymal stem cells (BMSCs) were co-cultured with different concentration of lithium chloride. Cell proliferation in each group was analyzed by 3-(4, 5-dimetyl-2-thiazoly-2, 5-diphenyl-2-H-tetrazolium bromide (MTT) assay. BMSCs were then co-cultured in osteogenic induction medium with different concentration of lithium chloride, and the expression of related mRNA was detected. The role of lithium in promoting BMSCs osteogenic differentiation and inhibiting BMSCs lipogenic differentiation was also investigated. Biomechanical properties of the tibia were evaluated at 8 weeks after operation. The tibial specimens of each group were collected at 4 and 8 weeks after surgery for histological examination and histological analysis. Micro-computed tomography (CT) scanning and 3D reconstruction were performed at 8 weeks. The results demonstrate that lithium can induce the osteogenic differentiation inhibit of adipogenic differentiation of BMSCs by regulating the Wnt signaling pathway. The histological evaluation further certified that average bone formation area in the group of tissue engineered HDPB doped with lithium was also significantly better than that of HDPB alone group. Based on the above evaluation, tissue engineered HDPB doped with lithium can effectively promote the regeneration of segmental bone defect, which can be used as a tissue engineering scaffold for clinical trials.

## Introduction

Repair of bone defects is a key step in orthopedic surgery and an inevitable problem in orthopedic surgery. In recent years, the number of bone defect patients significantly increased due to the traffic accidents and the incidence of bone tumors^1,2^. Therefore, a large number of bone grafts are urgently needed in clinical practice, yet it is still difficult to obtain adequate bone substitutes^3^. Autologous bone grafts can provide osteoblasts and bone-induced growth factors, providing similar tissue structure as local bone, which is considered as gold standard for bone defect repair. However, the sources of autologous bone are limited, and it usually causes pain in the donor site and requires additional surgery^4^. In order to obtain alternative materials with similar characteristics, researchers have focused on allograft and xenograft^5^, most of which have similar structure and properties to autologous bone, but these substitutes have poor intrinsic bone induction ability. In order to improve their osteogenic effect, additional improvements are needed to these materials^6^.

HDPB is an immunogenic heterogeneous bone extracted from the femur or tibia of pigs. It is mainly composed of hydroxylapatite (HA) and collagen. It owns porous mesh structure and good biocompatibility^7,8^. Due to the poor osteogenic effect, many studies have focused on improving its osteogenic induction performance^5^. Growth factors were commonly used to modify HDPB scaffolds, among which bone morphogenetic protein-2 (BMP-2) was the most commonly used protein to increase the osteogenic effect of HDPB^9^. Other growth factors including platelet -derived growth factor (PDGF), vascular endothelial growth factor (VEGF), and erythropoiesis (EPO) have also been attempted to be added to HDPB to improve bone conduction performance^10–12^. The addition of ions with special activities on the surface of scaffold is a new method of bone repair. Park et al.^13^ reported that magnesium doped pig bone with composite surface nanostructure could rapidly promote new bone formation in rabbit skull bone defects. However, scientific reports on these approaches are still limited.

Lithium (Li) has been used as psychoactive drug in clinic for half a century, it has also been reported to increase bone density^14^. Several studies have reported that lithium can promote osteogenic differentiation of BMSCs by activating the Wnt/GSK-3 signaling pathway^15,16^. In vivo studies demonstrated that lithium has promoting effect on bone regeneration and can improve osteoporosis^17,18^. In recent studies, lithium salts have been introduced into bone tissue engineering and have been shown to play an important role in improving the osteogenesis of biomaterials^19,20^. This study aims to investigate the osteogenic improvement effect of tissue engineered HDPB doped with lithium in vitro and in vivo, and evaluate its effectiveness in the healing of bone defects.

## Materials and methods

### Animals

In this study, 90 healthy adult female SD rats, aged 4 weeks and weighing 200–250 g, were provided by the Experimental Animal Center of West China Clinical Medical College, Sichuan University. 80 rats were used to construct bone defect models, while the others were used for the isolation and culture of BMSCs. All animal experiments conducted in this study have been approved by the Animal Management and Use Committee of West China Clinical Medical College of Sichuan University (Approval Number: SCXK20150012). All methods are implemented in accordance with relevant guidelines and regulations. The study was carried out in compliance with the ARRIVE guidelines. The rats were placed in cages 1 week before the experiment to adapt to the environment. 3 animals/cages were provided sufficient conventional animal feed to maintain the environment at 21 °C, 60% air humidity and 12 h circadian rhythm.

### Effect of different lithium concentrations on the proliferation of BMSCs

Cells from third generation in good growth condition were digested by trypsin and mixed into 1 × 10^5^/mL cell suspension. The cells were inoculated on 12-well plates and cultured at 37 °C with 5%CO_2_ saturated humidity. BMSCs were co-cultured with different concentration gradients of lithium (0, 2, 4, 6, 8, 10, 12, 14 mM), the time of cell number doubling was counted periodically. MTT assay was used to analyze cell proliferation in each group. Cells in the logarithmic growth stage and in good growth state were taken, the cell density was adjusted to 3 × 10^5^/ml, and the cells were inserted into a 96-well plate with 100 μl cell suspension in each well. Meanwhile, a blank group was set as control and cultured overnight at 37 °C (100 μl sterile PBS was added into the holes around the well). Cells were treated according to different groups and cell treatment settings, with 3 poly pores in each group and cultured at 37 °C for 24 h. 10 μl MTT was added into each well and cultured at 37 °C for 4 h. The medium was sucked out and 150 μl DMSO was added and shock for 10 min. The absorbance value of each well was determined by microplate reader.

### Effect of lithium concentration on osteogenic differentiation of BMSCs

Cells of the third generation in good growth condition were used and digested by trypsin, and the cells were mixed into 1 × 10^5^/ml cell suspension. The cells were inoculated on the 12-well plate and cultured at 37 °C with 5%CO_2_ saturated humidity. After the cells were completely adherent to the wall for three days, BMSCs were co-cultured with different concentration gradients of lithium in osteogenic induction medium. The osteogenic induction medium was replaced every three days. After 21 days, ALP stain was performed for identification of differentiated osteoblasts and the optical density of ALP positive staining in each group was measured with Image-Pro Plus 6.0 software. The expressions of osteocalcin and ALP in each group were detected by semi-quantitative RT-PCR.

### Wnt signaling pathway in promoting osteogenic differentiation of BMSCs by lithium

Cells were inoculated on the 12-well plate and cultured at 37 °C with 5%CO_2_ saturated humidity. Cells were cultured for three days until they completely adherent to the wall. In order to explore the mechanisms of LiCl induced ossification, BMSCs were cultured in osteogenesis medium (IMDM with 10% FBS, 5 μg/ml insulin, 0.1 μM dexamethasone, vitamin C and 0.2–10 mM β glycerol phosphate) containing lithium chloride (12 mM). At the same time, DKK and DKK + LiCl were added as the control group, BMSCs were cultured in osteoblast induction culture alone as the blank control group, osteoblast induction culture solution was replaced every three days, ALP stain was performed after 14 days and the optical density of ALP positive staining in each group was measured. The expression multiplication of RUNX2, a marker gene in Wnt signaling pathway, was detected by RT-PCR.

### Wnt signaling pathway in inhibition of lipid differentiation of BMSCs by lithium

Cells of the third generation were mixed into 1 × 10^5^/mL cell suspension. The cells were inoculated in the 12-well plate. Cells were cultured for three days until they completely adherent to the wall. BMSCs were cultured in lipopolysaccharide- inducing medium (IMDM medium with 10% FBS, 10 μg/ml insulin, 1 μM dexamethasone, 0.5 mM IBMX, 0.1 mM indomethacin) containing lithium chloride (12 mM). At the same time, DKK and DKK + LiCl were set as control group, BMSCs were cultured in lipopolysaccharide induction medium as blank control. The culture medium was changed every three days. Oil red O staining was performed after 14 days and the optical density of lipid droplets in each group were measured. The mRNA expression of PPARG, a marker of lipid differentiation, was detected by RT-PCR.

### BMSCs were inoculated on HDPB scaffolds

The prepared HDPB scaffolds were sterilized with 75% ethanol for 24 h, then washed with PBS for 3 times, and soaked in BMSCs medium (DMEM) for 1 day before inoculation. The excess medium on the scaffolds was removed, and BMSCs suspension (5 × 10^6^/ml) was slowly and carefully inoculated on the porous scaffolds. The prepared composites were incubated in an incubator containing 5%CO_2_ and 37 °C for 3 h, and then incubated in an environment of 100% humidity and 37 °C for 7 days. The medium was replaced every 48 h.

### Preparation of tibial defect model and stent implantation

SD rats were anesthetized by intraperitoneal injection of 10% chloral hydrate (0.3 ml/100 g), the left hind limb was shaved, and sterilized with alcohol. A posterior medial tibial incision was made to expose the tibia by blunt dissection of subcutaneous tissue and muscular layer. A 5 mm bone defect was created using a bone saw and the corresponding graft material was implanted according to the groups. The rats were randomly divided into 5 groups (16 in each group). In the control group, bone defect wasn’t filled and was just fixed with intramedullary nails. HDPB scaffold was implanted into the bone defect site in the HDPB group, and 5 × 10^6^/ml BMSCs were injected into the bone defect site in the BMSCs group. The HDPB + BMSCs group was inoculated with 5 × 10^6^/ml of BMSCs and implanted into the bone defect site. In the HDPB + LiCl + BMSCs group, 5 × 10^6^/ml BMSCs and 12 mM LiCl HDPB biological scaffolds were implanted into the bone defect site. After the bone defect was fixed with intramedullary needles, the muscle and skin were sutured layer by layer. All operations were performed under aseptic conditions. Dressing of the wound was changed every day, the wound and needle orifice were sprayed with iodine volt, and penicillin was intramuscularly injected at 40 MU/day. Tibia was collected at 4 and 8 weeks, respectively, and fixed in 10% formaldehyde solution for further analysis.

### Biomechanical test

Eight weeks after surgery, 6 rats were taken from each group to evaluate the biomechanical properties of the affected limb tibia. Specimens were collected, soft tissues were taken out, and biomechanical properties of segment stiffness (N/mm) and ultimate load (N) of bone defects were measured by RGT-5a. Ultimate load test: the moving speed of the beam was 2 mm/min, the precision of load measurement was 0.01 mm, and the load-deformation curve was recorded by the recorder. Stiffness test: the moving speed of the beam is 5 mm/min, the constant deflection is 1 mm, and the bending failure curve is recorded; Data are plotted as mean ± standard deviation. The mean value of all SD rats on the non-operative side was taken as the control group as the evaluation standard and compared with the measured values on the experimental side.

### Histological detection and histomorphological analysis

Rats were sacrificed at 4 and 8 weeks after surgery, and tibial specimens were taken. The specimens were fixed with 10% formalin and decalcified with EDTA. The tissue was dehydrated and fixed with graded ethanol (80–100%) and embedded in paraffin. Embedded specimens were cut into 5 µm sections and stained with hematoxylin and eosin (HE) and Masson’s trichrome. Specimens were examined under a BX53 microscope. Image-pro Plus 6.0 software was used to detect the size of new bone and the number of new blood vessels. The area of newly formed bone is normalized as a percentage of the new bone area divided by the total defect area. The density of neovascularization was measured by dividing the number of neovascularization vessels by the size of the defect. Ten samples were detected in each group, and five sections were taken from each sample for measurement.

### Micro-CT detection

In order to evaluate the newly formed mineralized tissue, micro-CT scan and 3D reconstruction of tibia were performed 8 weeks after surgery. According to the method described in the literature^21^, the percentage of newly formed mineralized bone volume in total tissue volume (BV/TV) and BMD were calculated.

### Statistical analysis

SPSS 21.0 statistical software was used for statistical analysis, and all data were expressed as mean ± standard deviation (${\bar{x}} \pm {\text{s}}$). Comparisons between groups were conducted by one-way analysis of variance (ANOVA), followed by Bonferroni or Dunnett posttest. *P* < 0.05 was considered statistically significant.

## Results

### Effects of different lithium concentrations on proliferation and osteogenic differentiation of BMSCs

**Figure 1:**
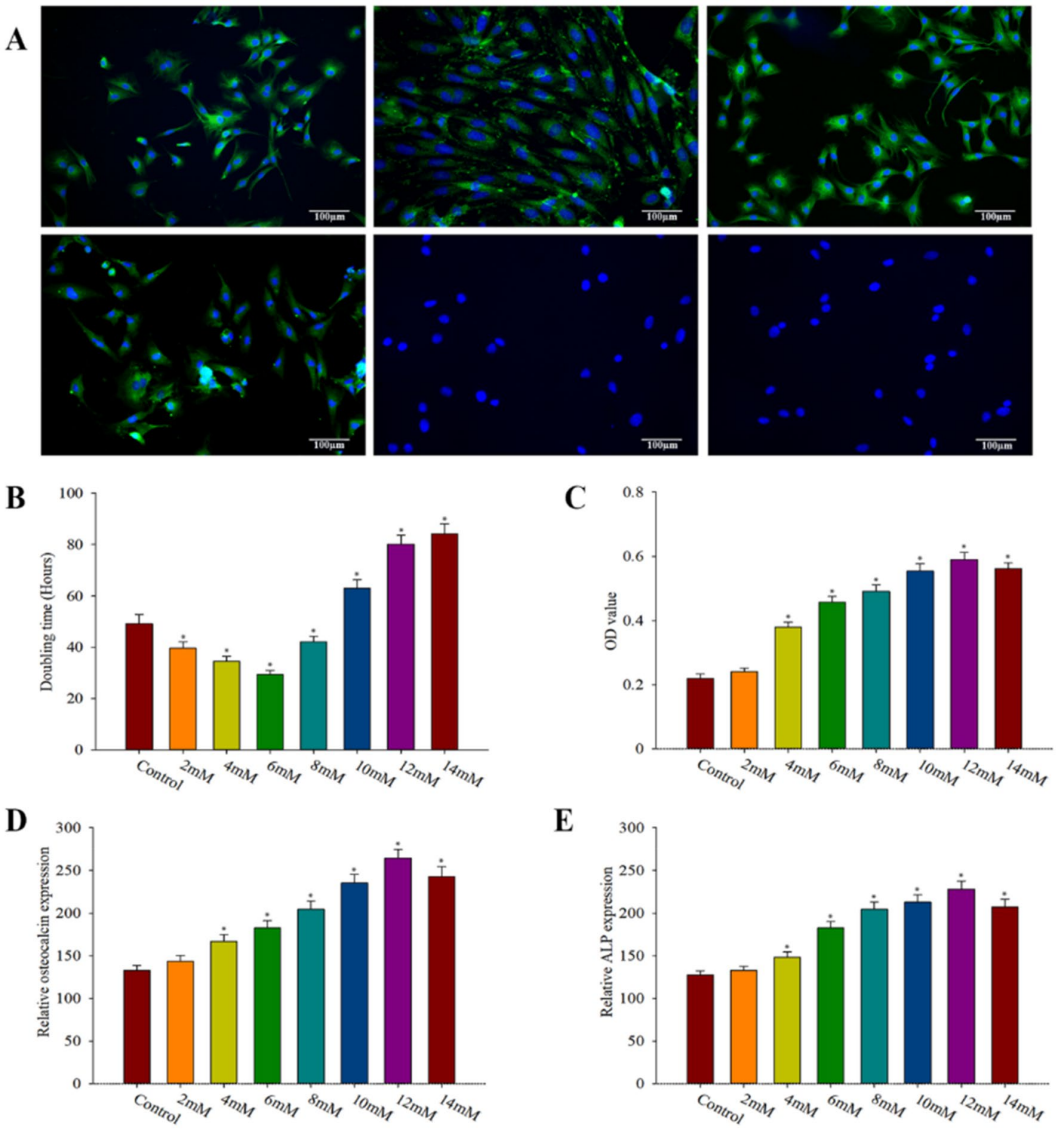
Effects of different lithium concentrations on BMSCs proliferation and osteogenic differentiation. (**A**) Characteristics of BMSCs. Results of immunofluores- cence labeling of third-generation bone marrow mesenchymal stem cells showed positive CD29 (**A**), CD44 (**B**), CD90 (**C**) and CD271 (**D**), and negative CD11b (**E**) and CD45 (**F**). (**B**) The multiplication time of BMSCs in the medium containing lithium salts of different concentration gradients, compared with the control group *P < 0.05; (**C**) Optical density (OD) of BMSCs cultured in osteogenic medium containing lithium salts of different concentration gradients for 21 days in ALP staining, compared with the control group *P < 0.05. (**D**) Expression of osteocalcin after BMSCs were cultured in osteogenic medium containing lithium salts of different concentration gradients for 21 days, compared with control group *P < 0.05. (**E**) ALP expression of BMSCs cultured in osteogenic medium containing lithium salts of different concentration gradients for 21 days compared with control group *P < 0.05.Three fields of view were taken for each group, and the data were plotted in the format of mean ± standard deviation.

Immunophenotype analysis (Fig. 1A) showed that CD29, CD44, CD90 and CD271 were strongly positive and CD11b and CD45 were negative, which was consistent with the characteristics of BMSCs. BMSCs doubling time was shortest when lithium salt concentration was 6 mM (29.39 ± 1.61 h), which decreased significantly compared with the control group (49.24 ± 3.60 h), the difference was statistically significant (*P* < 0.0001) (Fig. 1B). With the increase of lithium concentration, the doubling time of BMSCs was prolonged. When the lithium concentration reached 10 mM, the doubling time of BMSCs (63.08 ± 3.06 h) was longer than that of the control group, and the difference was statistically significant (*P* < 0.05). After 21 days of culture in osteogenic induction medium, ALP stain was performed, and ALP positive staining were observed in all groups (Fig. 1C). The optical density values of each group were measured (three fields were selected for each group) and statistically analyzed. The results showed that, with the increase of lithium concentration, the optical density values of ALP positive staining gradually increased. The optical density of ALP positive staining was the highest (0.59 ± 0.02) when the lithium concentration was 12 mM, which was significantly increased compared with the control group (0.22 ± 0.01), and the difference was statistically significant (*P* < 0.01). Later, as the concentration of lithium increased, the degree of osteoblast differentiation decreased. According to the semi-quantitative RT-PCR results, relative expression of osteocalcin was closely related to lithium concentration, reaching its peak as lithium concentration was 12 mM, which significantly increased compared with the control group, there are significant difference statistically significant (*P* < 0.01). Afterwords, the expression of osteocalcin decreased with the increase of lithium concentration (Fig. 1D). The expression trend of ALP was similar to that of osteocalcin (Fig. 1E). The relative expression in the 12 mM lithium concentration medium was (228.20 ± 9.33), which was significantly increased compared with the control group (127.54 ± 4.94), and the difference was statistically significant (*P* < 0.01).

### Lithium induces osteogenic differentiation while inhibits lipid differentiation of BMSCs by regulating Wnt signaling pathway

**Figure 2:**
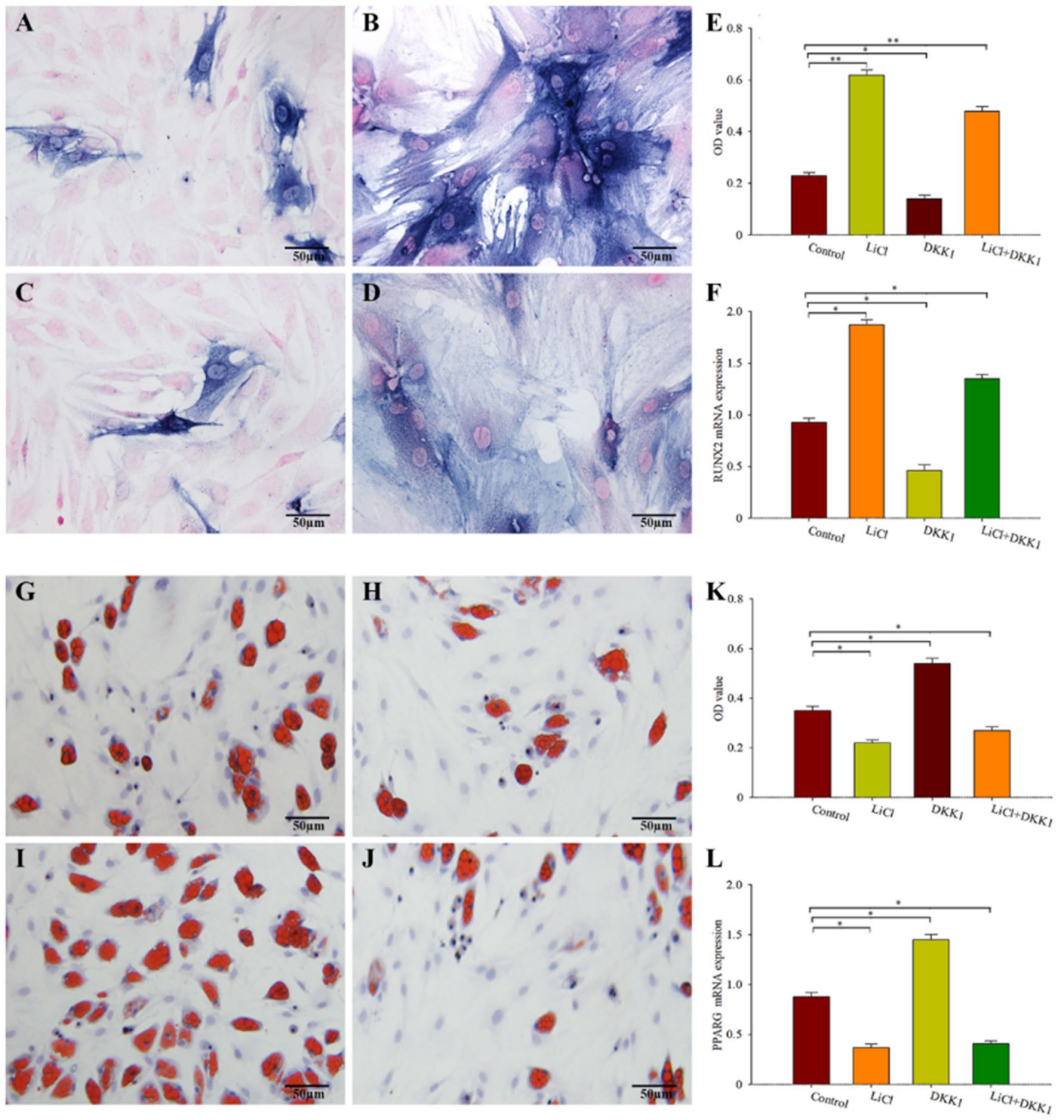
Lithium induces osteogenic differentiation of BMSCs by regulating Wnt signaling pathway. (**A**) Bone BMSCs were positively stained with ALP after 14 days of culture in osteogenic medium (magnification: 400 times). (**B**) The number of ALP positive staining of BMSCs increased significantly after 14 days of cultivation in osteogenic medium containing LiCl (12 mM) (magnification factor: 400 times). (**C**) DKK1 significantly inhibits the osteoblast differentiation (magnification: 400 times); (**D**) LiCl was added to the medium and ALP positive staining were increased again (magnification factor: 400). (**E**) The results of statistical analysis of the optical density of ALP positive staining in the four groups were: *P < 0.05, **P < 0.01, compared with the control group. (**F**) Expression multiplication of marker gene RUNX2 in Wnt signaling pathway was detected by RT-PCR, *P < 0.01 compared with the control group. (**A**) BMSCs were positive for oil red O staining after 14 days of culture in adipogenic medium, forming characteristic red lipid droplets (magnification: 400 times). (**B**) The number of lipid droplets formed by BMSCs cocultured with LiCl decreased significantly (magnification: 400 times). (**C**) DKK1 significantly increases lipid droplet formation (magnification: 400 times). (**D**) LiCl was added to the medium and a small amount of lipid droplets could be observed (magnification: 400 times). (**E**) Statistical analysis results of optical density values of lipid droplets in each group, *P < 0.01 compared with the control group. (**F**) mRNA expression of PPARG was detected by RT-PCR, *P < 0.01 compared with the control group. Three fields of view were taken for each group, and the data were plotted in the format of mean ± standard deviation.

To investigate the mechanism of LiCl inducing osteogenic differentiation of BMSCs, BMSCs were cultured in osteogenic medium (IMDM with 10% FBS, 5 mg/ml insulin, 0.1 mg dexamethasone, 0.2 mM vitamin C and 10 mM beta-glycerine phosphate). As shown in Fig. 2A, intracellular ALP positive staining can be seen, suggesting that BMSCs cultured in osteoblast induction medium can effectively differentiate into mature osteocytes. BMSCs were treated with osteogenic medium containing lithium chloride (12 mM) for 14 days, the number of ALP positive staining increased significantly compared with control group (Fig. 2B,E), with significant difference (*P* < 0.01). As a typical Wnt signaling pathway inhibitor, DKK1 significantly inhibited the ALP positive staining (Fig. 2C,E). Interestingly, after DKK1 treatment of BMSCs, LiCl was added to the medium and ALP positive staining increased again (Fig. 2D,E). In addition, RT-PCR was used to detect the mRNA expression of RUNX2, a marker gene for osteogenic differentiation, and the results showed (Fig. 2F) that co-culture with LiCl could significantly increase the expression of RUNX2 in BMSCs, with statistically significant difference compared with the control group (*P* < 0.01). DKK1 reduced RUNX2 expression, which was statistically significant compared with the control group (*P* < 0.01). These results suggest that lithium can induce osteogenic differentiation of BMSCs by regulating Wnt signaling pathway.

As mentioned above, BMSCs differentiation into bone cells and fat cells, in order to explore the LiCl between BMSCs to the dispersion of fat, we will between BMSCs in a concomitant induction medium (IMDM medium with 10% FBS, 10 mu g/ml insulin, 1 mM dexamethasone, IBMX 0.5 mM, 0.1 mM indomethacin) in the training, and oil red O staining. As shown in Fig. 2G, after 14 days of culture, the oil red O staining was positive, indicating the differentiation of bone marrow mesenchymal stem cells into lipids. The number of lipid droplets formed by BMSCs co-cultured with LiCl decreased significantly Fig. 2H,K), and the difference was statistically significant compared with the control group (*P* < 0.01). In contrast, DKK1 significantly increased lipid droplet formation (Fig. 2I,K), with statistically significant differences compared to the control group (*P* < 0.01). A small number of lipid droplets were observed in the LiCl + DKK1 group, indicating that the LiCl site is downstream of DKK1 (Fig. 2J,K). For further verification, RT-PCR was used to detect the mRNA expression of PPARG, the marker gene of adipogenic differentiation, and the results showed (Fig. 2L) that co-culture with LiCl could significantly reduce the expression of PPARG, and the difference was statistically significant compared with the control group (*P* < 0.01). DKK1 increased the expression of PPARG, which was statistically significant compared with the control group (*P* < 0.01). These results suggest that LiCl inhibits the differentiation of BMSCs into lipids by acting on the Wnt signaling pathway.

### Histological analysis of bone defect regeneration

**Figure 3:**
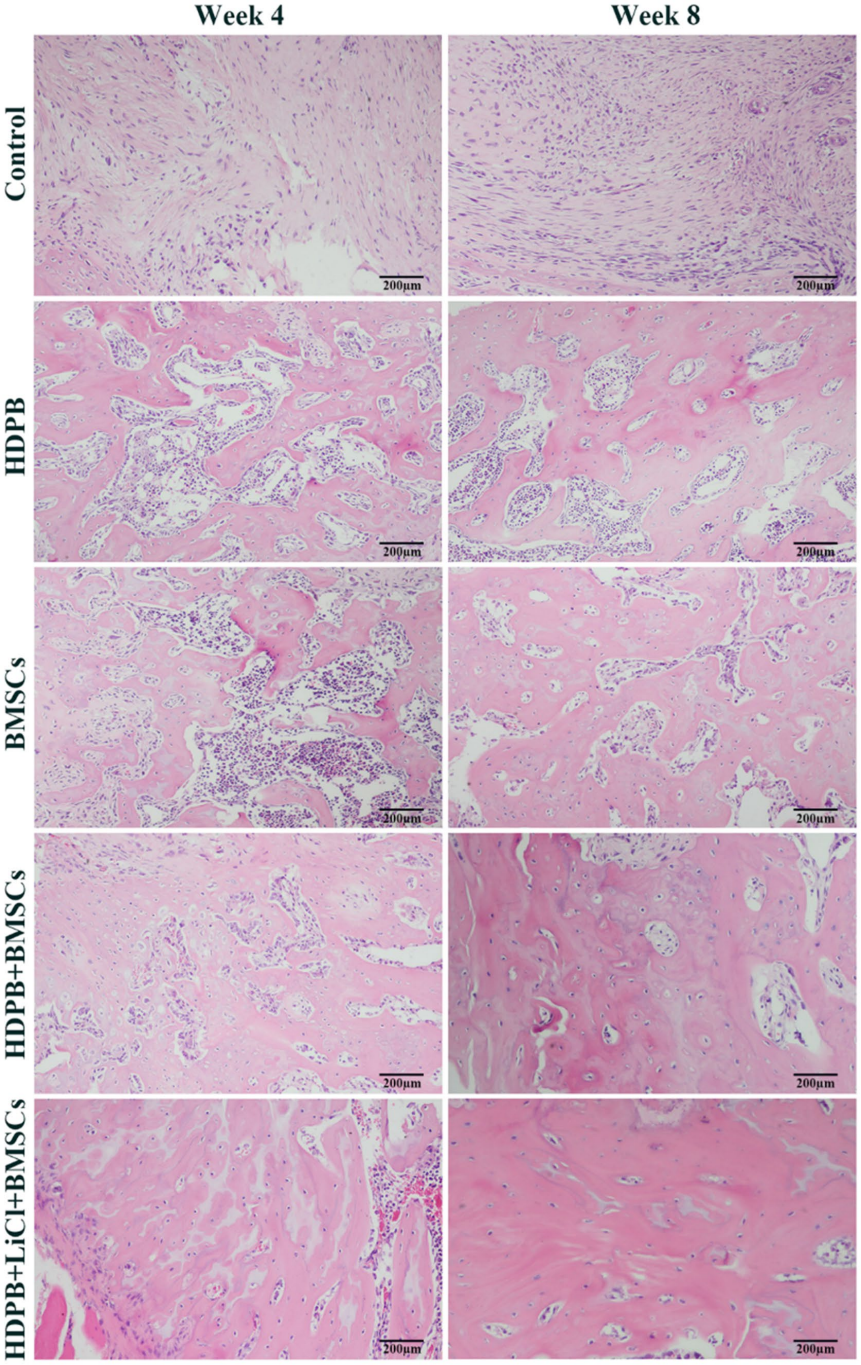
Representative HE staining images of each group at 4 and 8 weeks after the surgery (200 × magnification). The new bone area is dyed pink.

**Figure 4:**
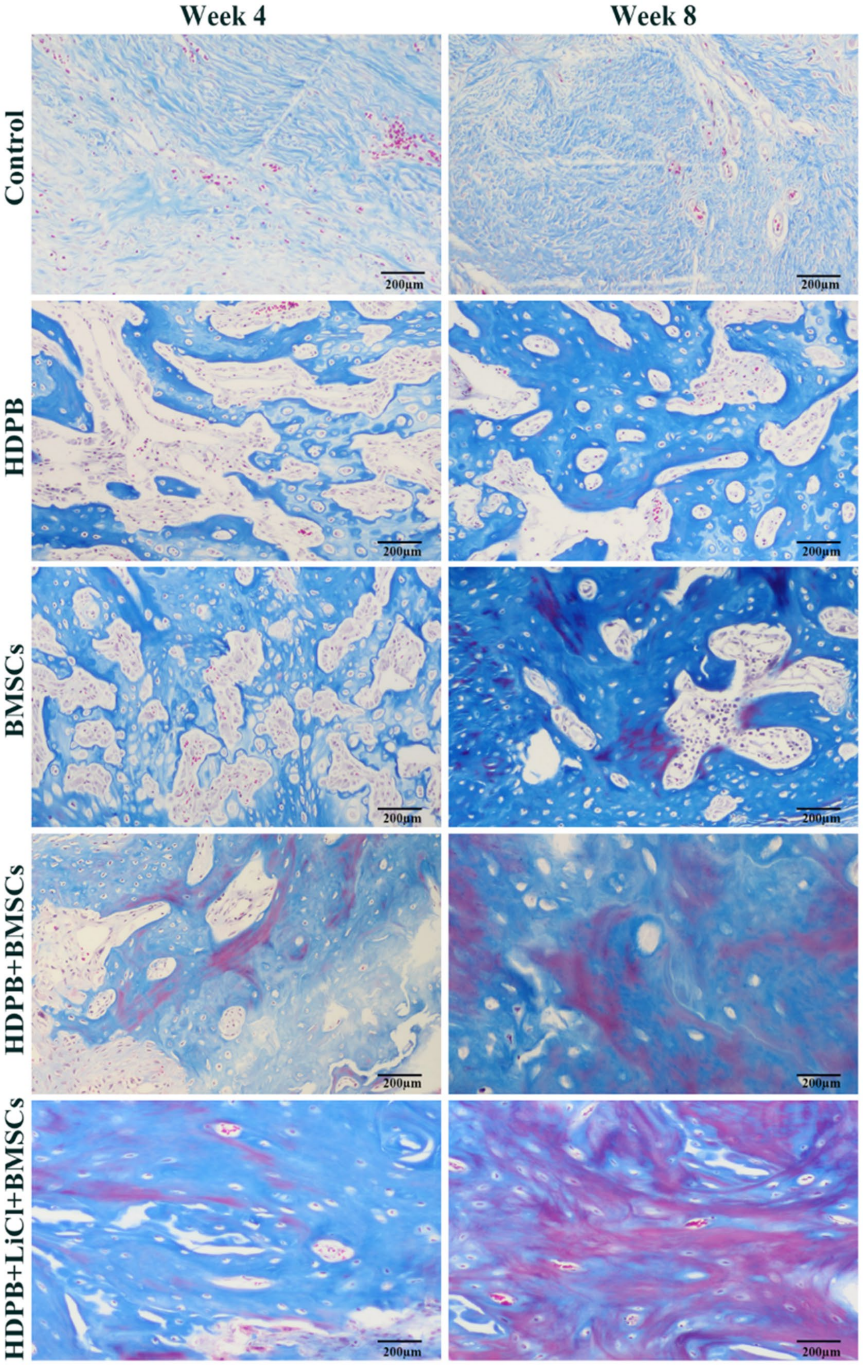
Representation of Masson's Trichrome staining (200 × magnification) at 4 and 8 weeks after surgery.

Histological analysis was performed at 4 and 8 weeks postoperatively to further investigate the effect of HDPB doped with lithium biological scaffolds on the repair of segmental bone defects. The representative H&E images of each group at different time points were shown in Fig. 3, the new bone was stained in dark red. At 4 weeks and 8 weeks, bone growth was vigorous in the defect areas of the HDPB + BMSCs group and the HDPB + LiCl + BMSCs group, and new blood vessels were observed in the bone regeneration area. However, staining images in the HDPB + LiCl + BMSCs group showed more new bone formation and more organized skeletal structures than those of other groups. In contrast, in the control group, there was only a small amount of new bone formation and more fibrous connective tissue in the defect area. Representative masson’s staining of new bone formation in each group was shown in Fig. 4. The regenerated bone in BMSCs group and HDPB group was immature with reddish staining, indicating that it was still in the early stage of mineralization. The HDPB + LiCl + BMSCs group had more new bone formation than the HDPB + BMSCs group. In addition, new bone in the HDPB + LiCl + BMSCs group was more mature and mineralized than those of other groups. No significant new bone formation was observed in the control group.

**Figure 5:**
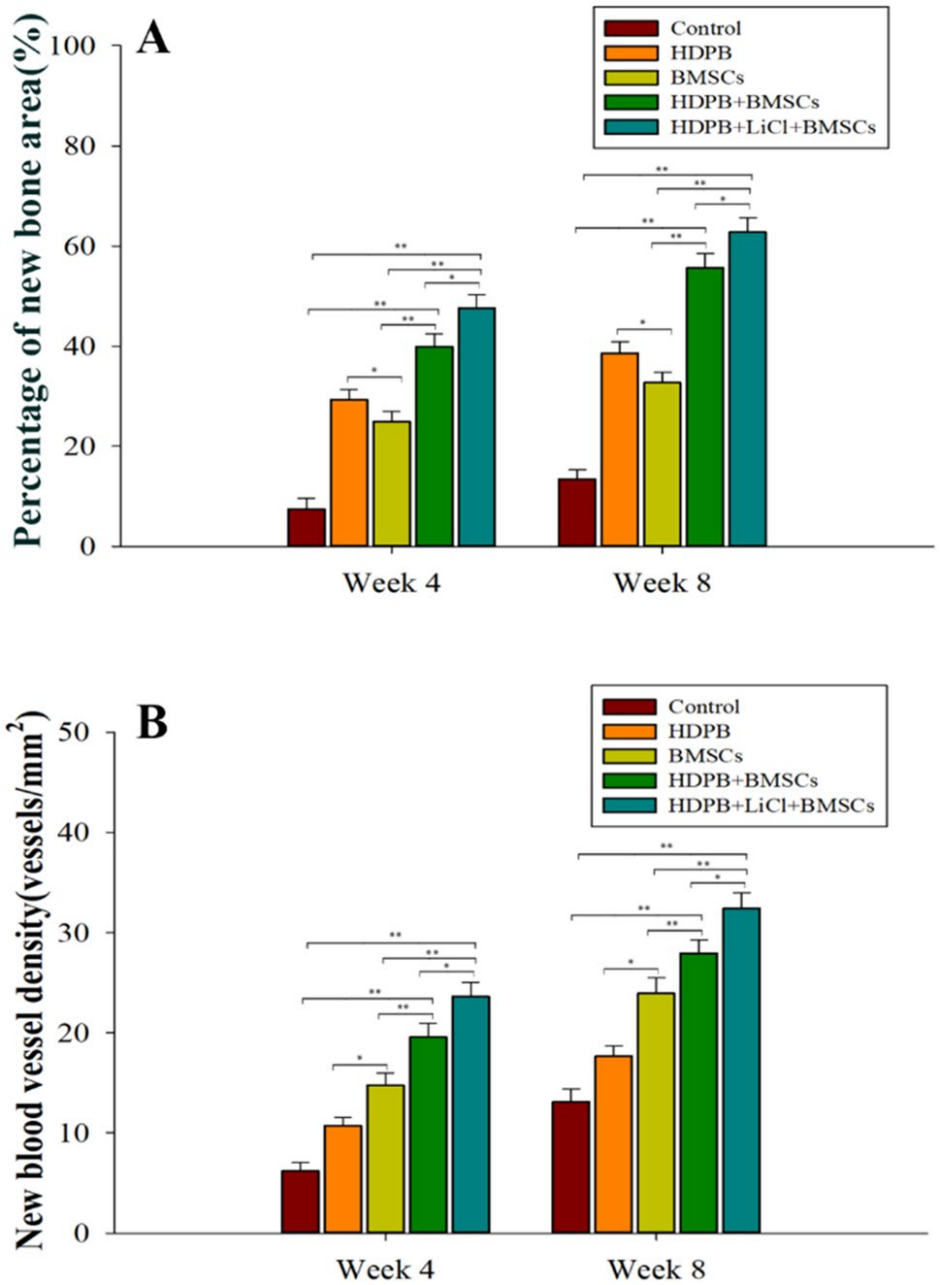
Quantitative evaluation of the bone repair ability of bone marrow mesenchymal stem cell biological scaffolds loaded with lithium-doped heterogeneous white bone. (**A**) The percentage of new bone volume in the bone defect area in each group at 4 and 8 weeks after the surgery, *P < 0.05, **P < 0.01. (**B**) The density of neovascularization in bone defect areas in each group at 4 and 8 weeks after the surgery, *P < 0.05, **P < 0.01. Three fields of view were taken for each group, and the data were plotted in the format of mean ± standard deviation.

The histomorphological methods were used to calculate the new bone area and the new vascular density, so as to quantitatively evaluate the ability of bone repair of lithium doped HDPB biological scaffolds. Comparison of new bone area in each group was shown in Fig. 5A. At all time point, the new bone area of HDPB + BMSCs group was significantly higher than that of the control group and BMSCs group, and the difference was statistically significant (*P* < 0.01). The new bone area of the HDPB + LiCl + BMSCs group was significantly higher than that of the HDPB + BMSCs group, and the difference was statistically significant *P* < 0.05). As can be seen from Fig. 5B, the new blood vessels density in the HDPB + BMSCs group was significantly higher than that in the BMSCs group and control group, with difference having outstanding statistical significance (*P* < 0.01). The density of new blood vessels in the HDPB + LiCl + BMSCs group was significantly higher than that in the HDPB + BMSCs group, there was great significance between them (*P* < 0.05).

### Biomechanical property of bone defect area

**Figure 6:**
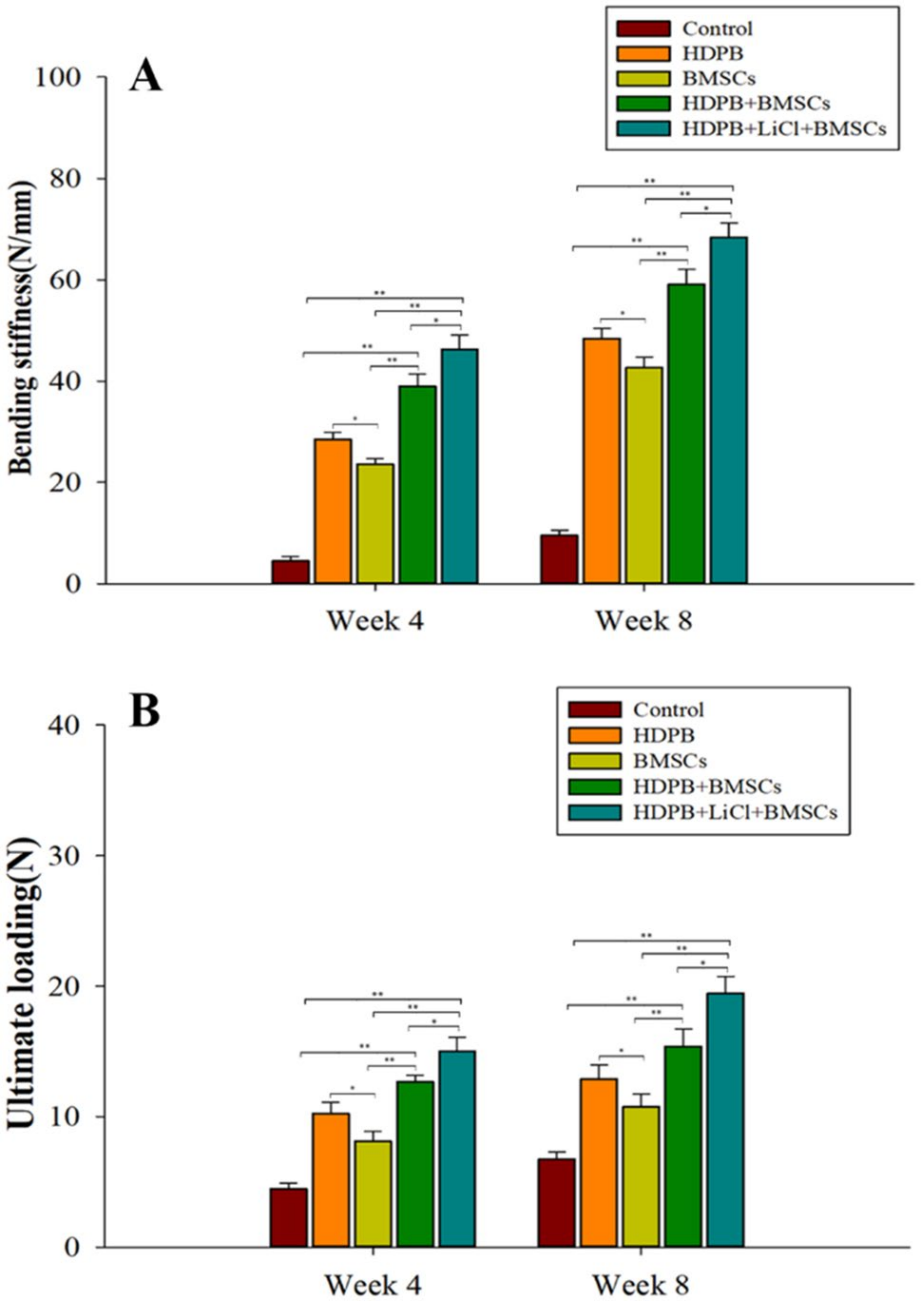
Evaluation of biomechanical changes in bone defect segments repaired with biomechanical scaffolds of bone marrow mesenchymal stem cells loaded with lithium-doped heterogeneous white bone. (**A**) Segment stiffness of bone defects in each group at 4 and 8 weeks after the surgery, *P < 0.05, **P < 0.01; (**B**) The ultimate load of bone defect segments in each group at 4 and 8 weeks after the surgery, *P < 0.05, **P < 0.01. Three samples were taken from each group for analysis, and the data were plotted in the format of mean ± standard deviation.

The biomechanical test results are shown in Fig. 6A,B. At the 4th week, the segmental stiffness and ultimate load values of bone defects in the HDPB + BMSCs group were significantly higher than those in the control group and the BMSCs group alone, with statistically significant differences (*P* < 0.01), and those in the HDPB + LiCl + BMSCs group were significantly higher than those in the HDPB + BMSCs group, with statistically significant differences (*P* < 0.05). The BMSCs group had lower segmental stiffness and ultimate load values than HDPB group, with statistically significant differences (*P* < 0.05). The biomechanical results at week 8 showed a similar trend to that at week 4. These results jointly indicated that the biomechanical strength of tibia at the segment of bone defect could be significantly enhanced by the biological scaffold of bone marrow mesenchymal stem cells loaded with lithium.

### Micro-CT evaluation of the bone defect area

**Figure 7:**
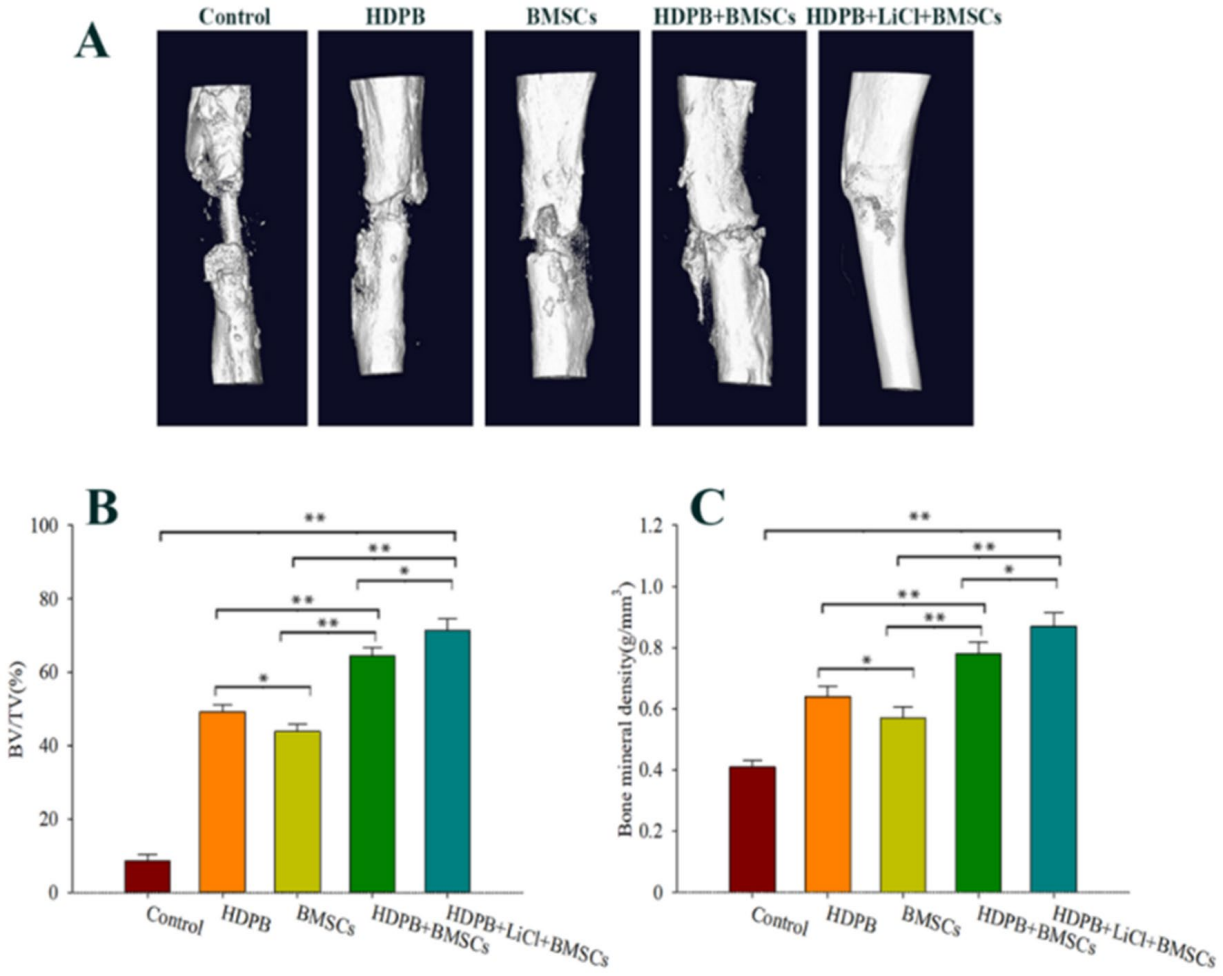
Micro-CT 3D reconstruction image and quantitative analysis results of bone defect area 8 weeks after the injury. (**A**) Representative three-dimensional reconstruction image at 8 weeks after the injury. (**B**) Eight weeks after the surgery, the percentage of new bone volume in each group of bone defect segments *P < 0.05, **P < 0.01. (**C**) Bone mineral density analysis results of bone defect segments in each group at 8 weeks after surgery, *P < 0.05, **P < 0.01. Five samples were taken from each group for analysis, and the data were plotted in the format of mean ± standard deviation.

At 8 weeks after injury, the bone volume repair in the defect area was evaluated in the long term. Micro-CT 3D reconstruction image showed (Fig. 7A) that there were more calluses in bone defect segments in the HDPB + BMSCs group than in the HDPB + BMSCs group, and there were more calluses in the HDPB + LiCl + BMSCs group than in the HDPB + BMSCs group. The control group had very little new bone formation. Figure 7B,C showed the percentage of new bone volume in total bone volume and bone mineral density in each defect segment. The percentage of new bone volume in total bone volume and bone mineral density of HDPB + BMSCs group were significantly higher than that of control group, HDPB group and BMSCs group at each time point, with statistically significant differences (*P* < 0.01). The percentage of new bone volume in total bone volume and bone mineral density of HDPB + BMSCs group were significantly higher than that of HDPB + BMSCs group (*P* < 0.05). The percentage of new bone volume and bone mineral density in the BMSCs group was lower than that in the HDPB group, and the difference was statistically significant (*P* < 0.05).

## Discussion

The purpose of this study was to evaluate the osteogenesis and repair of bone defects of tissue engineered HDPB doped with lithium. In our in vitro study, the presence of tissue engineered HDPB doped with lithium was beneficial to the proliferation and calcium deposition of bone marrow mesenchymal stem cells. In animal experiments, The tissue engineered HDPB doped with lithium performed better in terms of the average gray value of X-ray, the average pixel value of CT, and the bone volume and trabecular thickness. These parameters helped to demonstrate that tissue engineered HDPB doped with lithium could improve the healing of critical bone defects. In addition, in the histological evaluation of new bone formation, the average area of bone formation in the HDPB + LiCl + BMSCs group was also significantly better than that of single HDPB group. Based on the above evaluation, the tissue engineered HDPB doped with lithium can promote the repair of bone defects.

The role of lithium in bone regeneration has been studied for many years. In previous studies, lithium has been shown to promote bone formation and improve osteoporosis^22^. In Clement et al.’s^23^ human experiments, lithium use in mice has been shown to significantly increase bone mass and bone density. In addition, Zammani et al.^15^ assessed hip and lumbar vertebral bone density in patients treated with lithium salts and found that lithium carbonate maintenance therapy could maintain or enhance bone mass. In animal models of steroid-induced osteoporosis, lithium can activate the Wnt signaling pathway by inhibiting GSK-3β activity, and reverse osteoporosis by improving osteoblast activity and inhibiting osteoclast mediated bone resorption^22^. Li et al.^24^ reported similar results that lithium ions released from lithium chloride/calcium phosphate bone cement could accelerate bone tissue regeneration in animals with osteoporosis by activating Wnt/β-catenin signaling pathway^17^. In fact, many studies have shown that lithium mainly promotes osteogenic differentiation and inhibits adipogenic differentiation by promoting the Wnt/beta-catenin signaling pathway in BMSCS^16,24–26^. However, there are few studies on the application of lithium in combination with exogenous bone substitutes in bone defect repair. Therefore, in this study, it is reasonable and significant to select lithium salt to improve the osteogenic effect of tissue engineered HDPB and apply its composite material to the repair of segmental bone defects.

Segmental bone defects are defined as intramuscular injuries that cannot heal spontaneously and completely without intervention^27^. In this study, a model of tibial defect in SD rats about 5 mm was selected, which has been proved to be a standardized model of bone defect in rats. The incorporation of surface bioactive ions into bone tissue engineering has been studied for many years^28^. Studies have shown that implants containing bioactive ions have sub-micron or nano-scale surface structure, which is conducive to more cell attachment and continuous release of bioactive ions into local tissues around the implants^29^. In this study, they chose a recognized scheme for biological coating of xenodesorption scaffold, and added lithium salt to the surface of xenodesorption scaffold. Their results showed that adding lithium salts to the surface of the heterogeneous deboning scaffold could cover the surface with nanoparticles, which, according to another studies^30^, could increase the contact area of the material and further improve the surface reactivity. In addition, since lithium salt is a kind of bioactive ion with osteogenic effect, our in vitro studies have shown that heterogeneous deboning with lithium has a positive effect on increasing calcium deposition. In the process of repairing segmental bone defects in vivo, lithium ions slowly released in the bone defect area will play a continuous role in bone induction. In our animal experiments, through imaging analysis and histological examination, it has been confirmed that lithium doped debone-removing stent can significantly promote the formation of new bone and the healing of bone defects, which has proved that lithium has positive effect on bone regeneration. Therefore, the preparation of lithium doped debone-removing stent is correct. After treatment with lithium-doped biofilm, trabeculae were reconstructed and segmental bone defects were almost healed. Meanwhile, PCR results showed that Runx2 was highly expressed in the cells.

Collectively, the present study demonstates that BMSCs of rats have a strong proliferation ability and can differentiate into osteoblasts under the induction of lithium, and express ALP and osteocalcin, which are suitable for bone tissue engineering seed cells. Tissue engineered HDPB doped with lithium can effectively promote the regeneration of bone tissue in bone defect segments and significantly improve the mechanical strength of the defected tibia, which can be used as a tissue engineering scaffold for clinical trials.
